# Experimental Investigation of Stress Distributions in 3D Printed Graded Plates with a Circular Hole

**DOI:** 10.3390/ma14247845

**Published:** 2021-12-18

**Authors:** Quanquan Yang, He Cao, Youcheng Tang, Yun Li, Xiaogang Chen

**Affiliations:** Jiangsu Province Key Laboratory of Advanced Manufacturing Technology, Huaiyin Institute of Technology, Huai’an 223003, China; caohe1@hyit.edu.cn (H.C.); tangyoucheng1@hyit.edu.cn (Y.T.); liyun@hyit.edu.cn (Y.L.); chenxiaogang1@hyit.edu.cn (X.C.)

**Keywords:** functionally graded plate, 3D printing, circular hole, stress analyses, experiment

## Abstract

An experimental investigation is presented for the stress distributions in functionally graded plates containing a circular hole. On the basis of the authors’ previously constructed theoretical model, two kinds of graded plates made of discrete rings with increasing or decreasing Young’s modulus were designed and fabricated in virtue of multi-material 3D printing. The printed graded plates had accurate size, smooth surface, and good interface. The strains of two graded plates under uniaxial tension were measured experimentally using strain gages. The stresses were calculated within the range of linear elastic from the measured strains and compared with analytical theory. It is found that the experimental results are consistent with the theoretical results, and both of them indicate that the stress concentration around the hole reduces obviously in graded plates with radially increasing Young’s modulus, in comparison with that of perforated homogenous plates. The successful experiment in the paper provides a good basis and support for the establishment of theoretical models and promotes the in-depth development of the research field of stress concentration in functionally graded plates.

## 1. Introduction

Functionally graded materials (FGMs) are a class of advanced composite materials in which the material properties change uninterruptedly along one or more directions. FGMs are characterized by a compositional gradient of one material into another, which is totally different from the conventional composite materials. The continuous change in microstructure of FGMs avoids the mismatch of material properties across the interface, and thus stress concentration can be effectively reduced compared with that existing at material interfaces [1,2]. Especially when the structures contain various holes, the stress concentrations around the holes can be decreased by choosing the properly radial changes of the elastic properties [3,4].

A lot of research has been carried out on the problem of stress concentration around holes in FGM plates with radially varying elastic properties in the past 20 years. Two analytical approaches to this problem are mainly employed. One is the numerical investigations on the basis of the finite element method. The other is rigorous theoretical analyses according to the linear elasticity theory.

In the aspect of numerical investigations, Venkataraman et al. [5] proposed a two-dimensional numerical model of the plate with a hole having a radially varying elastic modulus, inspired by the special material distributions near the blood vessel holes in bone. They explained how bones design holes by optimizing the structure of the perforated plates with biological variables. To reduce stress concentration and increase load-carrying capacity of the plate with holes, Huang et al. [6,7] also optimized the material distribution near the hole by mimicking bones through axisymmetric and nonaxisymmetric FGMs. On the basis of the finite element method, Kubair and Bhanu-Chandar [8] and Wang et al. [9] studied the stress concentration coefficient (SCF) in FGM plates with circular and elliptical holes, respectively, under uniform tensile traction. The elastic properties varying along three directions (X, Y, and radial direction) are discussed. Goyat et al. [10,11] analyzed the reduction of SCF around a rectangular hole and a pair of circular holes in homogenous plates by using a FGM layer based on the extended finite element method. Berezhnoi et al. [12,13] presented the exact solutions of stress distribution in the graded layers of super flywheel and discussed the effect of the relative radius of the holes in the flywheel on the specific energy.

In the aspect of theoretical analyses, Zhang et al. [14] proposed an analytical model of radial FGM plates with a circular hole and derived exact thermal stress solutions for the plates under axisymmetric thermal loading. Yang et al. [15,16] developed the theoretical models for the cases of non-axisymmetric loads, including arbitrary uniform tension and elastic wave. They decomposed the continuous FGM plates into a homogeneous plate containing multi-rings with different elastic constants and then solved the elastic fields on the basis of complex function theory. Mohammadi et al. [17] presented the general solution of SCF around a circular hole in a radially inhomogeneous plate under uniaxial tension, biaxial tension, and pure shear. Sburlati et al. [18] proposed a method to relieve the SCF around holes in homogeneous plates by using radial FGM ring and presented an analytical solution of SCF, as Young’s modulus varies with a monotonic power law. Kubair [19,20] considered the SCF and stress-gradients due to a circular hole in a radial FGM plate under anti-plane shear and presented the exact expressions for the elastic fields. Nie et al. [21] studied the problems of material tailoring for reducing SCF at circular holes in a FGM plate. Nie and Batra [22] further studied the reduction of SCF in homogeneous plates with holes by inserting a functionally graded incompressible material layer.

However, few experiments have been performed on the topic compared with present theoretical and numerical investigations. It is well known that the experiments play a fundamental and supporting role in scientific research. The first-hand experimental data can provide basis and support for the establishment of theoretical models and promote the in-depth development of the corresponding research field. The activities in the experimental research on stress concentration in FGM plates are limited mainly by the complexity and difficulty of the manufacturing technology of specimens. Buskirk et al. [23] experimentally studied the strength of a FGM biomimetic plates with a hole, which is designed based on the functional gradation in elastic modulus properties, as observed in the bone. The FGM plate was fabricated by different foam types, such as cellular, solid, and high density. Combined with finite element analyses, they verified that the FGM biomimetic plate has a higher load carrying capacity than a homogenous plate. In this paper, we present the experimental investigation for the stress distributions in graded plates fabricated by multi-material 3D printing. The surface morphology of the printed plates was observed by optical microscope. The objective of this paper is to present the design, fabrication, analysis, and results from mechanical tests of FGM plates with a circular hole and compare the results to the theoretical predictions given by the authors’ previously theoretical model.

## 2. Theoretical Model

**Figure 1:**
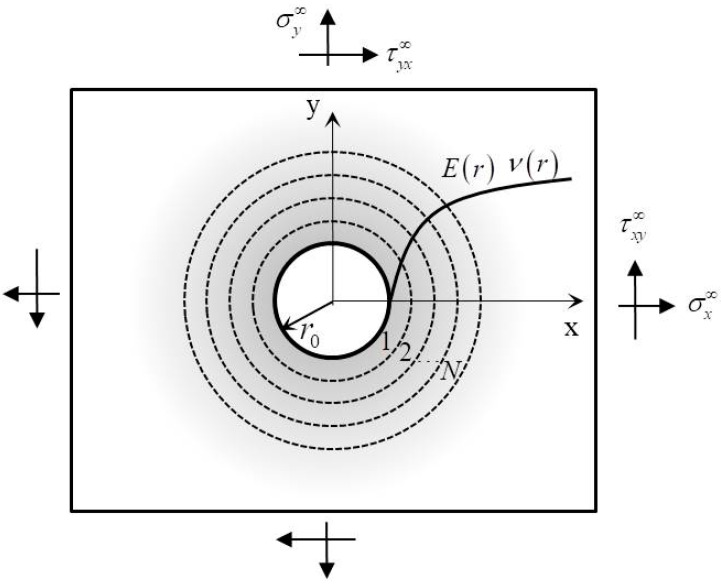
The theoretical model of a FGM plate with a circular hole developed in the authors’ previous work [15].

In the authors’ previous work [15], the theoretical model was developed for the stress concentration in an infinite FGM plate with a circular hole subjected to remote uniform loads $\sigma_{x}^{\infty }$, $\sigma_{y}^{\infty }$, and $\tau_{xy}^{\infty }$, as shown in Figure 1. $\sigma_{x}^{\infty }$, $\sigma_{y}^{\infty }$, and $\tau_{xy}^{\infty }$ are the normal stress and shear stress at infinity, respectively. The radius of the hole is denoted by $r_{0}$. The elastic properties are assumed to change continuously and arbitrarily in the radial direction. With the method of piece-wise homogeneous layers, the domains of FGM plate can be decomposed into N homogeneous rings $\Omega^{\left(1\right)},\Omega^{\left(2\right)},\cdots \Omega^{\left(j\right)},\cdots \Omega^{\left(N\right)}$ with equal width and an outer homogeneous plate. When the number of rings N is large enough, the radially continuous Young’s modulus E(r) and Poisson’s ratio ν(r) is approximately regarded as constant $E^{j}$ and $\nu^{j}$(j=1,2,⋯N) in each ring. Therefore, the boundary condition of force and displacement at the surface of the hole and the interface between the rings $\Omega^{\left(j\right)}$ and $\Omega^{\left(j+1\right)}$ can be expressed as

(1) $$X_{n}=Y_{n}=0,$$

(2) $$X_{n}^{\left(j\right)}=-X_{n}^{\left(j+1\right)},\;Y_{n}^{\left(j\right)}=-Y_{n}^{\left(j+1\right)},$$

(3) $$u^{\left(j\right)}=u^{\left(j+1\right)},\;v^{\left(j\right)}=v^{\left(j+1\right)},$$

where $X_{n}$, $Y_{n}$, u, and v symbolize the components of force and displacement.

Based on the theory of the complex variable functions, the boundary conditions for a plane problem of a homogeneous solid in a fixed rectangular coordinate system ($x{,}_{}y$) can be expressed as [24]

(4) $$\varphi \left(z\right)+z\bar{\varphi^{\prime }\left(z\right)}+\bar{\psi \left(z\right)}=\pm i\int_{0}^{s}\left(X_{n}+iY_{n}\right)ds,\;z=x+iy,$$

(5) $$\kappa \varphi \left(z\right)-z\bar{\varphi^{\prime }\left(z\right)}-\bar{\psi \left(z\right)}=2G\left(u+iv\right),$$

where φ(z) and ψ(z) stand for the potential functions. κ and G are elastic constants with different values for the cases of plane stress and plane strain.

According to Equations (4) and (5), Equations (1)–(3) can be expressed as

(6) $$\varphi_{1}\left(z\right)+z\bar{\varphi_{1}{}^{\prime }\left(z\right)}+\bar{\psi_{1}\left(z\right)}=0,$$

(7) $$\varphi_{j}\left(z\right)+z\bar{\varphi_{j}{}^{\prime }\left(z\right)}+\bar{\psi_{j}\left(z\right)}=\varphi_{j+1}\left(z\right)+z\bar{\varphi_{j+1}{}^{\prime }\left(z\right)}+\bar{\psi_{j+1}\left(z\right)},$$

(8) $$\frac{1}{G_{j}}\left[\kappa_{j}\varphi_{j}\left(z\right)-z\bar{\varphi_{j}{}^{\prime }\left(z\right)}-\bar{\psi_{j}\left(z\right)}\right]=\frac{1}{G_{j+1}}\left[\kappa_{j+1}\varphi_{j+1}\left(z\right)-z\bar{\varphi_{j+1}{}^{\prime }\left(z\right)}-\bar{\psi_{j+1}\left(z\right)}\right],$$

The complex potential functions in each circular ring $\Omega^{\left(j\right)}$(j=1,2,⋯N) and the plate can be expressed as [24]

(9) $$\varphi_{j}\left(z\right)=\sum_{-\infty }^{\infty }a_{-k}^{\left(j\right)}{\left(\frac{R_{0}}{z}\right)}^{k},\;\psi_{j}\left(z\right)=\sum_{-\infty }^{\infty }b_{-k}^{\left(j\right)}{\left(\frac{R_{0}}{z}\right)}^{k},$$

(10) $$\varphi_{N+1}\left(z\right)=\tau z+\sum_{k=0}^{\infty }a_{-k}^{\left(N+1\right)}{\left(\frac{R_{0}}{z}\right)}^{k},\;\psi_{N+1}\left(z\right)=\tau^{\prime }z+\sum_{k=0}^{\infty }b_{-k}^{\left(N+1\right)}{\left(\frac{R_{0}}{z}\right)}^{k},$$

where $a_{-k}^{\left(j\right)}{,}_{}b_{-k}^{\left(j\right)}$ $a_{-k}^{\left(N+1\right)}{,}_{}b_{-k}^{\left(N+1\right)}$ are the unknown coefficient and $R_{0}$ is a reference radius. τ and $\tau^{\prime }$ are the constants dependent on the remote stresses as $\tau \;=\;\left(\sigma_{x}^{\infty }+\sigma_{y}^{\infty }\right)/4$, $\tau^{\prime }\;=\;\left(\sigma_{y}^{\infty }-\sigma_{x}^{\infty }\right)/2+i\tau_{xy}^{\infty }$.

Substituting Equations (9) and (10) into Equations (6)–(8), we can get a set of linear equations containing all unknown coefficients $a_{-k}^{\left(j\right)}$, $b_{-k}^{\left(j\right)}$, $a_{k}^{\left(j\right)}$, $b_{k}^{\left(j\right)}$ and $a_{-k}^{\left(N+1\right)}$, $b_{-k}^{\left(N+1\right)}$. These equations can be applied to determine these unknown coefficients. In this case, the general solution of the stress fields in each ring and the homogenous plate can be derived based on the following field equations for the two-dimensional problem of the solid [24]

(11) σy+σx=4Re[φ′(z)],

(12) $$\sigma_{y}-\sigma_{x}+2i\tau_{xy}=2{\left[\bar{z}\varphi^{\prime \prime }\left(z\right)+\psi_{}{}^{\prime }\left(z\right)\right]}_{},$$

where $\sigma_{x}{,}_{}\sigma_{y}$, and $\tau_{xy}$ are the components of stresses.

In the work [15], authors discussed the effect of different functions of the Young’s modulus on the stress distribution in the plate. On the base of analysis and comparison, the desired optimal distribution of Young’s modulus in the selected functions is the following exponential form:

(13) $$E\left(r\right)=E_{0}\left(1-0.8\frac{e}{e^{r/r_{0}}}\right),$$

where $E_{0}$ is a constant. As the Young’s modulus changes in the above function, there is no stress concentration around the hole, and the stress distribution is almost uniform in the plate.

## 3. Experimental Procedures

In this paper, the experiment was designed and conducted based on the theoretical model. Two kinds of FGM plates made of discrete rings were fabricated for the cases of Young’s modulus, increasing and decreasing along the radial direction in virtue of multi-material 3D printing. The PolyJet 3D Printer named Stratasys Objet260 Connex3 (Stratasys Ltd., Rehovot, Israel) was used for printing the specimens of the FGM plate. This 3D Printer had a system size of 870 × 735 × 1200 mm and maximum build size of (XYZ) 255 × 252 × 200 mm. The printing accuracy reached up to 0.2 mm for full model size, and minimum build layer thickness could be as fine as 16 μm.

### 3.1. Tension Test of the Printing Materials

**Figure 2:**
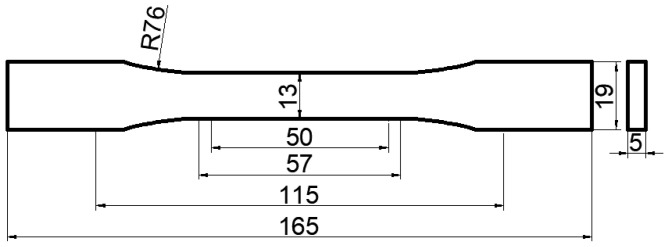
Dog-bone-shaped specimen for tension test (ASTM D638-14).

Two types of base materials (VeroWhitePlus and TangoBlackPlus, Stratasys Ltd., Rehovot, Israel) were used by the 3D Printer. VeroWhitePlus was rigid at room temperature and made from isobornyl acrylate, acrylic monomer, urethane acrylate, epoxy acrylate, acrylic monomer, acrylic oligomer, and a photoinitiator. TangoBlackPlus was rubbery at room temperature and formed from urethaneacrylate oligomer, Exo-1,7,7-trimethylbicyclo hept-2-yl acrylate, methacrylate oligomer, polyurethane resin, and photoinitiator [25,26]. The 3D printer mixed two base materials to form a broad range of intermediate materials with different strengths, which were predefined by the manufacturer. Six materials were selected to design and fabricate the required FGM plate for the experiment. Material 1 was the first base material VeroWhitePlus. Materials 2–6 were intermediate materials with graded strengths. The manufacturer only reported Young’s modulus of VeroWhitePlus as E = 2000–3000 MPa. In order to obtain the specific Young’s moduli of six materials, their tensile properties were tested according to the Standard Test Method of Plastics D638-14 made by the American Society for Testing Material (ASTM). The dog-bone-shaped specimen for tension test in the Standard is shown in Figure 2 [27].

**Figure 3:**
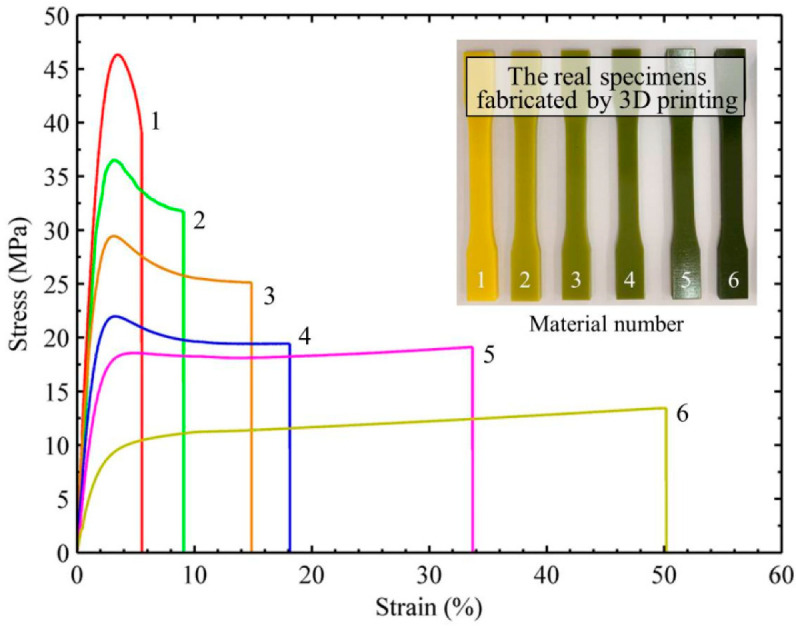
Stress-strain curves for six printing materials in tension.

The 3D models of the tension specimens were firstly built with commercial software Solidworks (sw2018, 2018, Concord, Massachusetts, America); then, with the models imported into the printing software Objet Studio, 3D objects could be automatically printed by 3D Printer. The six obtained specimens can be found in Figure 3. The tensile properties of specimens were tested by electronic universal testing machine. Stress-strain curves are shown in Figure 3. It can be found that the strength of material 1 was the highest and material 6 was the lowest. The strengths of material 2–5 were between those of material 1 and 6. The Young’s moduli of six printing materials were also measured and listed in Table 1. It is found that six Young’s moduli showed a well-graded change, which was in accordance with our expectation. In the following experiment, the specimens of FGM plates were designed and fabricated with using six printing materials.

### 3.2. Design and Fabrication of FGM Plate with a Circular Hole

**Figure 4:**
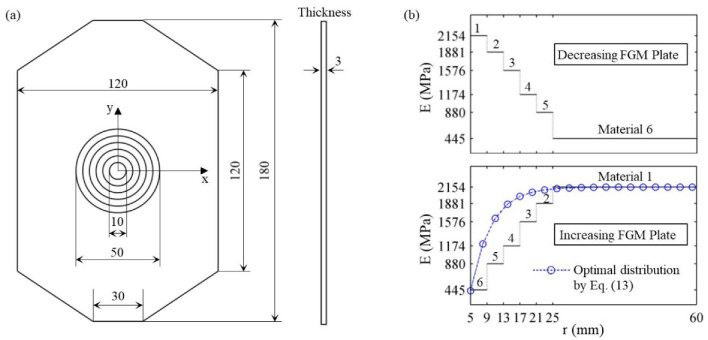
Design for FGM plates with a circular hole: (**a**) geometric design (mm), (**b**) material design.

On the basis of the theoretical model in Section 2, two FGM plates with the same geometrical sizes but different material distribution were designed, as shown in Figure 4. In terms of geometric design in Figure 4a, the width and thickness of the FGM plate were taken as 120 mm and 3 mm, respectively. The diameter of the center hole was 10 mm. There were five rings, and the width of each ring was 4 mm. The width of the plate was more than 10 times the diameter of the hole. According to the results shown in Figures 10 and 11 in the paper by Yang et al. [28], who discussed the effect of FGM plate size on the stress concentration, the plate can be approximately considered infinite in the case and it causes few errors, which can be neglected. Therefore, the following experimental results for the designed plates can be compared with the theoretical solution, which was derived based on the assumption of the infinite plate in Section 2.

In terms of material design, two different cases, in which Young’s modulus decreases and increases in the radial direction, were designed, as shown in Figure 4b. The materials in the five rings and outer plate were set in the order of materials 1-6 and 6-1, respectively. They are named the decreasing FGM plate and increasing FGM plate, respectively, throughout the paper. During the design of increasing FGM plate, we matched the desired optimal distribution of Young’s modulus given by Equation (13) as best we could, given the available materials.

**Figure 5:**
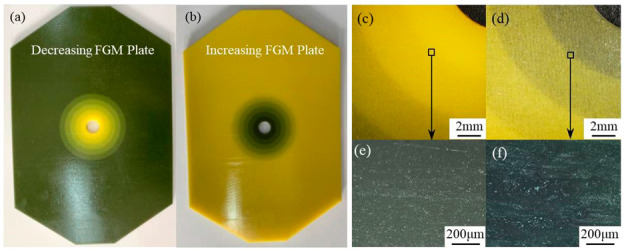
Specimens of two FGM plates fabricated by 3D printing: (**a**) photo of decreasing FGM plate, (**b**) photo of increasing FGM plate, (**c**) macromorphology of decreasing FGM plate, (**d**) macromorphology of increasing FGM plate, (**e**) interface morphology of decreasing FGM plate, (**f**) interface morphology of increasing FGM plate.

In order to fabricate two FGM plates, 3D models were firstly built by Solidworks, according to the geometric sizes of two FGM plates. It is worth emphasizing that in order to set different material types in the software of 3D Printer, each part with different material in the plate should be separately built during the modeling process. The complete 3D model of FGM plate was obtained by assembling each separate part. The specimens of FGM plate were then fabricated, similar to the printing process of previous tension specimen. The obtained specimens are shown in Figure 5. According to different material colors in Figure 5a,b, it can be found that the material distributions in two FGM plates were in accordance with material design. Figure 5c,d shows the surface macromorphology of two FGM plates observed by microscope DSX 100 (Olympus Corporation, Tokyo, Japan). It is clear that the surface was smooth and there were no macroscopic cracks, inclusions, or other defects in two plates. Figure 5e,f shows the interface morphology observed by Carl Zeiss Axio Imager (Carl Zeiss AG, Oberkochen, Germany). It is found that two FGM plates had high density, good bonding interface, and forming quality.

### 3.3. Strain Measurement of FGM Plates

In this work, strain gages were utilized to determine the strain distribution along the radial direction in the FGM plate. The physical dimensions of strain gauges were 3.5 × 2.2 mm. Seven measuring points were chosen, and their radial positions on the *x*-axis are listed in Table 2. The points with numbers #1–5 were located in five rings, and points #6 and #7 were located in the outer plate. For the two-dimensional plane stress problems in the paper, stress calculation required knowledge of normal strain in both *X* and *Y* directions. Therefore, two strain gages were pasted parallel to the *X* and *Y* axis, respectively, for each measuring position. Moreover, in order to monitor and minimize the out-of-plane warping deformation of the plate after fabrication or any other possible out-of-plane bending during loading, a further seven groups of strain gages were mounted on the back faces of the plate corresponding to each measured position. Two strain gauges with the same radial position on two faces were connected to the half bridge circuit in the experiment.

**Figure 6:**
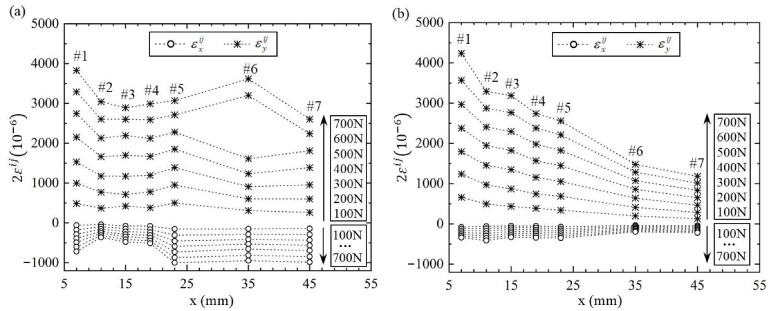
The measured strains $\varepsilon_{}^{ij}$ along the *x*-axis in two FGM plates: (**a**) decreasing FGM plate, (**b**) increasing FGM plate.

The incremental loading method was employed in the tension test. The load was increased with the same scale as ΔF = 100 N each time and from 100 N up to 700 N in seven loads. The strains $\varepsilon_{x}^{}$ and $\varepsilon_{y}^{}$ at seven measured points were read out for each load level, as listed in Table 3 and Table 4. Since the measurement was made on the half bridge connection, the original measured strains in Table 3 and Table 4 are two times the real strain. The real strains in the paper were expressed as $\varepsilon_{}^{ij}$, where the superscript i represents the loading times and the j represents the number of measured points. It is worth noting that all materials in the whole FGM plate were within the range of linear elastic deformation under the maximum tensile load 700 N. In order to show the measured strains more visibly, the variations of the strains $\varepsilon_{}^{ij}$ along the *x* direction were drawn, as in Figure 6 for two FGM plates under seven different loads. It can be seen that as the loads increase, the strains $\varepsilon_{x}^{ij}$ and $\varepsilon_{y}^{ij}$ at each measured point decrease or increase with equal magnitude. In particular, two strains $\varepsilon_{y}^{ij}$ for the loads F = 600 N and 700 N fluctuated obviously at point #6 in the decreasing plate. The reason for fluctuations may be the instability of resistance strain gauge or the problem of bridge balance during measurement.

## 4. Results and Discussions

### 4.1. Theoretical Results

The theoretical solution of the stress field was firstly presented via the analytical method [15] before discussing the experimental results. According to the dimensions of printed FGM plates, we took the radius of circular hole as $r_{0}\;=\;5\;\mathrm{mm}$ in the theoretical model and decomposed the FGM plate into five homogenous rings in the domain r = 5~25 mm and an outer homogenous plate. The Young’s moduli $E^{j}$(j = 1,2,⋯5) in the rings and $E^{6}$ in outer plate were chosen as those listed in Table 1. Moreover, the remote loading stresses were taken as $\sigma_{x}^{\infty }=0$, $\tau_{xy}^{\infty }=0$, and

(14) $$\sigma_{y}^{\infty }=F_{i}/A,$$

where the cross-sectional area of the plate is $A{\;=\;360\;\mathrm{mm}}^{2}$. Since the variation of Poisson’s ratio has little influence on the stress distribution [15], Poisson’s ratio in the whole plate is assumed to be a constant ν(r) = 0.3, which is the common value for materials. 

**Figure 7:**
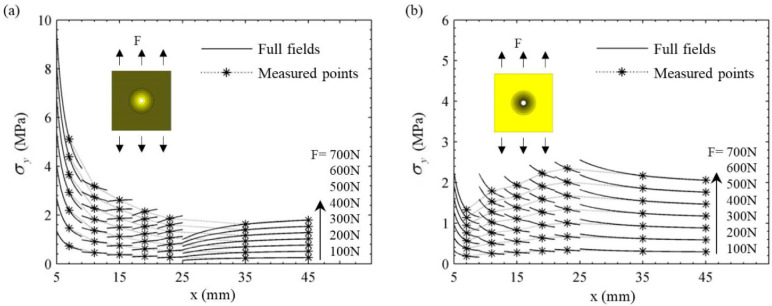
Theoretical results of stresses $\sigma_{y}$ along the *x*-axis in two FGM plates: (**a**) decreasing FGM plate, (**b**) increasing FGM plate.

The theoretical results of stress $\sigma_{y}$ in two FGM plates under seven load levels are shown in Figure 7. The solid lines represent the full field stress distributions. In order to make a more intuitive comparison with the experimental results, the theoretical results of stress at the measured points are marked with asterisks in Figure 7. It can be seen that the full-field stress in two FGM plates increased in a regular manner, with the load increasing from 100 N to 700 N. Due to the difference of Young’s modulus between each ring, the hoop stress had an obvious abrupt change at the interface of each ring. By comparing the results in Figure 7a,b, it can be found that the stresses $\sigma_{y}$ of two FGM plates differ small away from the hole but differ greatly near the hole. For the decreasing FGM plates, the stresses near the hole were very high and about 4–5 times of those in the far field. On the contrary, for the increasing FGM plates, the stresses near the hole were very low and even lower than those in the far field.

### 4.2. Experimental Results

Based on the experimental strains $\varepsilon_{x}^{ij}$ and $\varepsilon_{y}^{ij}$ in Table 3 and Table 4, the stresses $\sigma_{y}^{ij}$ at seven measured points can be calculated according to the generalized Hooke’s law due to the tensile deformation within the range of linear elastic

(15) $$\sigma_{y}^{ij}=\frac{E^{j}}{1-\nu^{2}}\left(\varepsilon_{y}^{ij}+\nu \varepsilon_{x}^{ij}\right),$$

where $E^{j}$ is Young’s modulus corresponding to each measured point.

**Figure 8:**
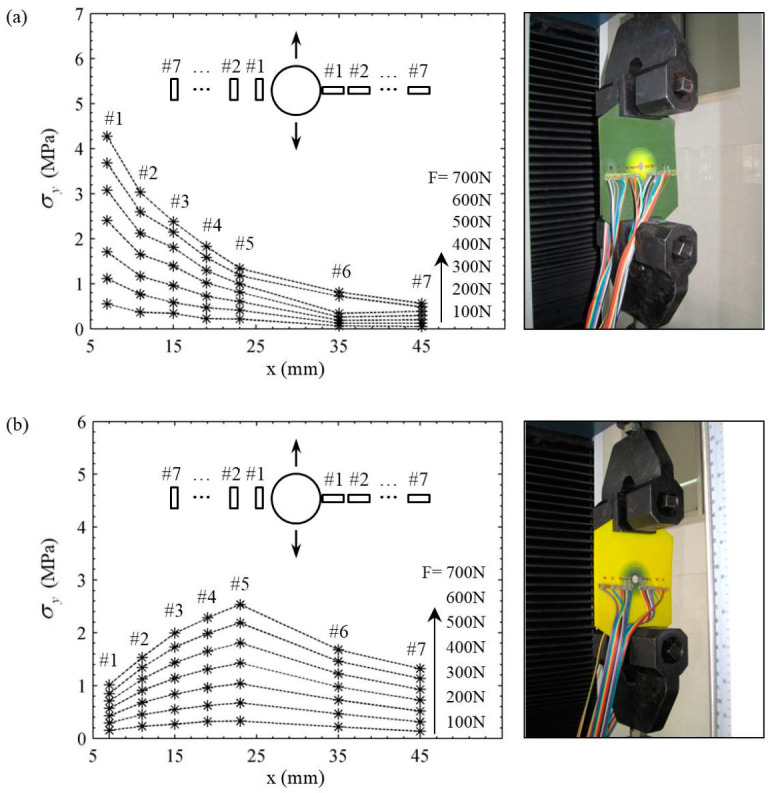
Experimental result of stresses $\sigma_{y}$ along the *x*-axis in two FGM plates: (**a**) decreasing FGM plate, (**b**) increasing FGM plate.

The variations of the experimental stress $\sigma_{y}$ along the *x* direction were drawn as in Figure 8 for two FGM plates under seven different loads. It can be seen that, as the load $F_{i}$ increased from 100 N to 700 N, the experimental stress at most positions in two plates increased by the same amount. In particular, there were certain fluctuations at the point of strain gage #6 in the decreasing plate in Figure 8a. It can be also found from Figure 8a that the stress value decreased rapidly away from the hole. The stresses near the hole were significantly higher than those in the far field. For the increasing FGM plate in Figure 8b, the stresses near the hole were lower than those in the far field, and the maximum stress appeared at the position of strain gage #5. The stress concentration did not occur at the edge of hole. The experimental results here were consistent with the theoretical analysis in Figure 7 and further prove an important conclusion proposed by Yang et al. [15]. That is, the decreasing Young’s modulus along the radial direction in the plate results in more severe stress concentration at the hole, while the increasing Young’s modulus can alleviate or even eliminate the stress concentration near the hole.

### 4.3. Comparison of Experimental and Theoretical Results

In order to make a comparison of experimental and theoretical results, their average stresses under seven loads were calculated. For the experimental results, we firstly calculated the strain increments $\Delta \varepsilon_{x}^{j}$ and $\Delta \varepsilon_{y}^{j}$, corresponding to the loading increment ΔF = 100 N at each point, and then calculated their average strain increments $\Delta \varepsilon_{x-\mathrm{ave}}^{j}$ and $\Delta \varepsilon_{y-\mathrm{ave}}^{j}$. Finally, the average stresses $\sigma_{y-\mathrm{ave}}^{j}$ can be derived at each point in the linear range

(16) $$\sigma_{y-\mathrm{ave}}^{j}=\frac{E^{j}}{1-\nu^{2}}\left(\Delta \varepsilon_{y-\mathrm{ave}}^{j}+\nu \Delta \varepsilon_{x-\mathrm{ave}}^{j}\right).$$

**Figure 9:**
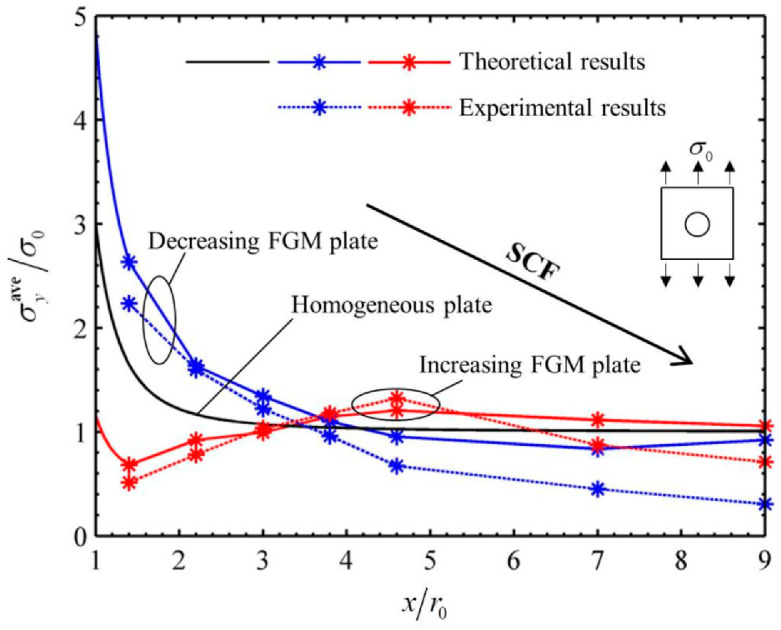
Comparison of experimental and theoretical results of dimensionless stresses $\sigma_{y}^{\mathrm{ave}}/\sigma_{0}$ along the *x* direction (the blue curve shows the decreasing FGM plate and red curve shows the increasing FGM plate).

The comparison of experimental and theoretical results of dimensionless hoop stresses along *x* direction is shown in Figure 9. The influences of the radius of a circular hole and loads were eliminated in the x-coordinate and y-coordinate, respectively. The referring stress $\sigma_{0}^{}$ in y-coordinate was taken as $\sigma_{0}^{}=\Delta F/A$. The black solid curve in Figure 9 denotes the stress distribution of a homogeneous plate containing a circular hole under uniaxial tension, which is one of the classical solutions in the elastic theory [24,25,26,27,28,29]. It is well known that the SCF is 3 for the case. The blue and red curves represent the stress distributions in decreasing and increasing FGM plates, and the theoretical and experimental results of two FGM plates are denoted by solid and dash lines, respectively. It can be found that, among three types of plates, the peak stress or SCF was highest in the decreasing FGM plate but lowest in the increasing FGM plate. It can also be found that, although the overall varying trend of experimental and theoretical results was consistent, three experimental points only of 14 points lay on the theoretically calculated curves. The other experimental points were more or less out of the theoretical predictions. 

The errors between the experimental and theoretical results of dimensionless stresses $\sigma_{y}^{\mathrm{ave}}/\sigma_{0}$ were calculated and listed in Table 5. It can be observed that the error was more than 15% at measured point #1 and more than 20% at points #6 and #7 in both plates. For measured point #1, the error was mainly caused by the limitation of the strain gauge method. As we all know, one strain gage can only measure one “point”, and the measured strain was just the average value measured over the active area of the strain gage. The active area of the strain gage covered a steep gradient of stress concentrations at the edge of hole (point #1). Therefore, its accuracy was much less. The errors at points #6 and #7 were mainly caused by the size of chucks in the testing machine. The width of the chucks was smaller than that of the plate, as shown in Figure 8. Therefore, the chucks only held the middle part of the plate during loading. The marginal area of the plate was not loaded enough. Therefore, the experimental stresses at points #6 and #7 were much lower than the theoretical predictions. The errors at other points mainly came from some assumptions in theoretical calculation and the operation of the experimental process; for example, (1) neglect of the variation of Poisson’s ratio in the theoretical analysis, (2) errors in the measurement of Young’s modulus of printing materials, (3) human operations, including mounting of strain gauge, clamping of specimen, etc. With the above factors taken into account, it can be considered that the experimental results verify the theoretical analysis within the reliable accuracy of the test.

## 5. Conclusions

Based on the authors’ previous theoretical model, two kinds of FGM plates containing holes with radially increasing and decreasing Young’s modulus were designed and fabricated by means of modern multi-material 3D printing. The strains at different radial points in two FGM plates were experimentally measured using strain gages under uniaxial tension. The stresses were calculated within the range of linear elastic from the measured strains. The results were compared with the theoretical solutions derived on the basis of the authors’ previously theoretical model. It is found that the experimental results agree with the theoretical solutions. They both demonstrate that the stresses near the hole in the decreasing FGM plate were much higher than that of homogeneous plate, while the stress concentration reduced obviously in the increasing FGM plate. The successful experiment and fabrication of the specimens in the paper are of great significance to the application of FGM in reducing stress concentration in the future.

## Figures and Tables

**Table 1:** Elastic modulus $E^{j}$ of six printing materials (MPa).

Material 1	Material 2	Material 3	Material 4	Material 5	Material 6
2154	1881	1576	1174	880	445

**Table 2:** Radial positions of seven measured points on the *x*-axis (mm).

Point #1	Point #2	Point #3	Point #4	Point #5	Point #6	Point #7
7	11	15	19	23	35	45

**Table 3:** Measured strains $2\varepsilon^{ij}$ ($10^{-6}$ ) in the decreasing FGM plate under different loads $F_{i}$.

**Load** $F_{i}$	Point #1		Point #2		Point #3		Point #4		Point #5		Point #6		Point #7	
	$2\varepsilon_{y}^{i1}$	$2\varepsilon_{x}^{i1}$	$2\varepsilon_{y}^{i2}$	$2\varepsilon_{x}^{i2}$	$2\varepsilon_{y}^{i3}$	$2\varepsilon_{x}^{i3}$	$2\varepsilon_{y}^{i4}$	$2\varepsilon_{x}^{i4}$	$2\varepsilon_{y}^{i5}$	$2\varepsilon_{x}^{i5}$	$2\varepsilon_{y}^{i6}$	$2\varepsilon_{x}^{i6}$	$2\varepsilon_{y}^{i7}$	$2\varepsilon_{x}^{i7}$
100 N	484.2	−59.2	370.2	−38.4	422.7	−75.1	379.2	−81.4	502.5	−153.7	309.7	−151.7	263.4	−142.9
200 N	997.4	−174	772.0	−96.2	714.5	−145.3	778.8	−163.9	951.3	−309.5	605.4	−287.9	602.5	−289.4
300 N	1530.1	−285.3	1177.3	−155.2	1173.5	−217.8	1192.3	−239.4	1389.2	−454.3	910.3	−414.1	955.5	−431.1
400 N	2151.3	−389.4	1662.5	−211.4	1698.7	−285.8	1669.3	−308.8	1854.2	−588.7	1235.7	−529.5	1387.3	−562.4
500 N	2746.2	−490.4	2129.4	−256.9	2193.6	−345.7	2122.2	−379.7	2281.4	−730.6	1610.7	−657	1812.1	−694.1
600 N	3290.3	−602.5	2605.7	−311.6	2601.2	−415.6	2589.7	−447	2712.9	−870.4	3200.5	−813.2	2239.6	−843.8
700 N	3828.0	−717.5	3043.2	−361.9	2891.7	−476.5	2989.0	−510.2	3072.8	−999.3	3619.9	−947.4	2604.9	−986.1

**Table 4:** Measured strains $2\varepsilon^{ij}$ ($10^{-6}$ ) in the increasing FGM plate under different loads $F_{i}$.

$\mathrm{Load}\;F_{i}$	Point #1		Point #2		Point #3		Point #4		Point #5		Point #6		Point #7	
	$2\varepsilon_{y}^{i1}$	$2\varepsilon_{x}^{i1}$	$2\varepsilon_{y}^{i2}$	$2\varepsilon_{x}^{i2}$	$2\varepsilon_{y}^{i3}$	$2\varepsilon_{x}^{i3}$	$2\varepsilon_{y}^{i4}$	$2\varepsilon_{x}^{i4}$	$2\varepsilon_{y}^{i5}$	$2\varepsilon_{x}^{i5}$	$2\varepsilon_{y}^{i6}$	$2\varepsilon_{x}^{i6}$	$2\varepsilon_{y}^{i7}$	$2\varepsilon_{x}^{i7}$
100 N	659.4	−78.1	496.5	−58.8	435.2	−49.2	388	−52.7	341.6	−74.2	198	−33.7	127.3	−39.9
200 N	1238.6	−121.9	971.4	−116.1	872.5	−92.7	746.3	−96.9	688.3	−120.9	410.7	−59.3	286.4	−69.2
300 N	1795	−170.1	1455.7	−174	1346.5	−141.2	1157	−148.7	1055	−165.9	640	−88.2	464.9	−98.3
400 N	2376.8	−212.3	1948.9	−234.9	1824.5	−189.2	1568.9	−194.5	1446.1	−214.6	860	−113.7	651.2	−129.9
500 N	2965.8	−258.1	2407.9	−294.3	2294.7	−235.4	1978.4	−242.7	1825.6	−260.9	1074.6	−139.2	833.5	−158.5
600 N	3567.6	−300.2	2876	−354.2	2764.9	−283.3	2380.8	−292.7	2210.6	−307.6	1285	−169.5	1014	−187.9
700 N	4238.4	−340.3	3293.6	−412.3	3187.2	−327.9	2740.5	−343	2557.1	−349.3	1476.9	−194.6	1182.6	−219.4

**Table 5:** The errors for experimental and theoretical results of dimensionless stresses $\sigma_{y}^{\mathrm{ave}}/\sigma_{0}$.

Point Number	Theoretical Result		Experimental Result		Error	
	Decreasing Plate	Increasing Plate	Decreasing Plate	Increasing Plate	Decreasing Plate	Increasing Plate
Point #1	2.631	0.682	2.234	0.514	−15.1%	−24.6%
Point #2	1.635	0.921	1.597	0.781	−2.3%	−15.2%
Point #3	1.342	0.995	1.220	1.033	−9.1%	3.8%
Point #4	1.101	1.146	0.960	1.177	−12.8%	2.7%
Point #5	0.954	1.207	0.672	1.323	−29.6%	9.6%
Point #6	0.835	1.114	0.451	0.874	−46.0%	−21.5%
Point #7	0.921	1.058	0.306	0.711	−66.8%	−32.8%

## Data Availability

Data are available on request to the corresponding author.
